# A flexible biomimetic superhydrophobic and superoleophilic 3D macroporous polymer-based robust network for the efficient separation of oil-contaminated water†

**DOI:** 10.1039/c9ra06579b

**Published:** 2020-01-31

**Authors:** Tawfik A. Saleh, Nadeem Baig, Fahd I. Alghunaimi, Norah W. Aljuryyed

**Affiliations:** Chemistry Department, King Fahd University of Petroleum & Minerals Dhahran 31261 Saudi Arabia tawfik@kfupm.edu.sa tawfikas@hotmail.com; Saudi Aramco, Research & Development Center, Oil & Gas Treatment R&D Division Dhahran 31311 Saudi Arabia; Saudi Aramco, EXPEC Advanced Research Center, Production Technology Division Dhahran 31311 Saudi Arabia

## Abstract

The development of stable 3D surfaces for oil/water separation has been of great interest to researchers. Inspired by the lotus leaf, in this study, a superhydrophobic stable and robust surface was generated by the combination of *n*-octadecyltrichlorosilane, silica, polypyrrole and polyurethane (ODTCS–SiO_2_–PP–PU). The constructed 3D network displayed superhydrophobic and superoleophilic behavior with a high water contact angle of 154.7° ± 0.8°. The superhydrophobic behavior of the porous material was found to be stable for months. Apart from the hydrophobicity analysis of the material, the various forms of the materials were investigated *via* scanning electron microscopy (SEM), Fourier-transform infrared spectroscopy (FTIR), and energy-dispersive X-ray spectroscopy (EDX). Under the force of gravity, hexane displayed an exceptionally high flux of 102 068 Lm^−2^ h^−1^ through ODTCS–SiO_2_–PP–PU. The macroporous network of ODTCS–SiO_2_–PP–PU displayed fewer chances of fouling, which is a common issue with membranes. Moreover, its porous network displayed good absorption capacity for various non-polar organic solvents. The maximum absorption capacity observed for toluene was 34 times its own weight. The separation efficiency of various non-polar organic solvents from water was observed in the range of 99.5 to 99.8%. ODTCS–SiO_2_–PP–PU, due to its superhydrophobicity, 3D porous network, extraordinarily high flux, good absorption capacity, and excellent separation capability, has been established as a good candidate for the separation of organic and oil contaminants from water.

## Abstract 2

Superhydrophobic polyurethane for oil and water separation.

## Graphical abstract

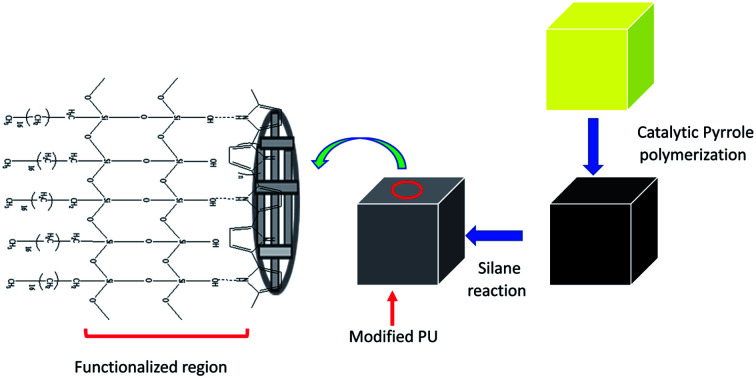

## 1. Introduction

Fast-growing urbanization and rapidly escalating global energy demands have increased crude oil exploration. Simultaneously, global energy demands also require a large-scale crude oil extraction and rapid offshore movement of oil. Developing new methods to remove oil from the water will potentially replace the conventional technologies.^1–4^ Several conventional methods have been applied for the separation of oil from water.^5^ The conventional methods include the *in situ* burning of spilled oil, mechanical skimming, flotation, and oil dispersion using a chemical dispersant. Conventional methods have some limitations, which are not economically favorable and also suffer from low separation efficiencies.^3^ In some cases, conventional methods are also a source of secondary pollutants. These demerits of the conventional methods make them unfavorable for the utilization of oil and water separation. This sort of limitation has motivated the scientific community to develop improved methods and materials that are more promising for the separation of oil and organic contaminants from water. These challenges can be addressed by introducing a superhydrophobic and superoleophilic porous surface.^6^ A variety of approaches that have been adopted to develop superhydrophobic surfaces include dip coating,^7^ temperature-based coating,^8^ lithography,^9^ chemical etching, sol–gel methods,^10^ templating,^11^ chemical vapor deposition, casting,^12^ electrospinning,^13^ and phase inversion methods. Various polymeric materials have been used to develop hydrophobic surfaces.^14^

Superhydrophobic surfaces that display a contact angle of greater than 150° have received a great deal of attention.^15^ There are two main characteristics that play a crucial role in the generation of superhydrophobic surfaces. The first factor is the surface roughness, which has a significant impact in improving the hydrophobicity. If a flat surface has a contact angle in the range of 100° to 120°, it may appear to be 150° to 170° on a microtextured or rough surface.^16^ The second factor that has a dominant role in the material hydrophobicity and hydrophilicity is surface energy. A low surface energy imparts hydrophobic behavior to the material. A material is superhydrophobic through the combination of low surface energy and the appropriate surface topography.^17^ In some cases, bumpy rough surfaces were generated by coating polymers on the porous surface and then, the surface was further coated with a low surface energy material to improve the hydrophobicity of the surface.^18^ On the basis of the specific wettability, various porous materials that can act as a filter or oil absorber were introduced.^19^ A material that displays the capacity to absorb or allow the passage of oil enables the collection of the oil. After the appropriate treatment, the collected organic component or oil can be reused. Although there has been extensive research in the field, there is still a remaining need to develop a material that is environmentally friendly, cost-effective, reusable, possesses a high absorption capacity, displays a high flux and is not a source of secondary pollutants.

Herein, a superhydrophobic interconnected porous network was constructed using a combination of polymer-based organic and inorganic materials. On the porous network of polyurethane, pyrrole was catalyzed into polypyrrole and then silica particles were introduced into the polypyrrole-coated polymeric walls of the polyurethane. The silica nanoparticles were furthermore functionalized with *n*-octadecyltrichlorosilane. As a result, the combination of polypyrene, polyurethane, silica, and *n*-octadecyltrichlorosilane provided a stable superhydrophobic surface that displayed a high flux and excellent absorption capacity. The developed porous materials displayed an excellent shelf life. The developed superhydrophobic network of ODTCS–SiO_2_–PP–PU displayed good reusability and it was used multiple times for flux studies along with the absorption of various organic solvents. The absorbed oil can be released through a simple process of squeezing. The high flux of 102 068 Lm^−2^ h^−1^ and excellent absorption capacity of 34 times its own weight has established a promising future for the superhydrophobic ODTCS–SiO_2_–PP–PU in the separation of oil and other organic contaminants from water.

## 2. Experimental

### 2.1 Materials

Pyrrole, *n*-octadecyltrichlorosilane, silicic-acid, hydrated ferric, chloride, ethanol, hexane, cyclohexane, heptane, isooctane, toluene, dodecane, polyurethane and *o*-xylene used for synthesis and testing of the materials were of analytical grade.

### 2.2 Instrumentation

Fourier transform infrared spectra of the various functionalized and non-functionalized samples were recorded using the Thermo Scientific Nicolet iS10 instrument. The surface morphology of the various samples was recorded using a scanning electron microscope (SEM) (JSM-6610LV) with 20 kV acceleration voltage. The uniform dispersion of the silicic acid was achieved by using a Derui® DR-P60 Ultrasonic Cleaner. The weight of the various chemicals was measured with a Mettler AE 200 weighing balance. The water contact angle on the surface of the various modified and unmodified PU was measured with the help of the Attension® Theta Biolin Scientific instrument.

### 2.3 Fabrication of biomimetic superhydrophobic network

The polyurethane foam was cut into small pieces (3 cm × 2 cm × 2 cm) and washed thoroughly to remove dirt particles from it. It was sonicated for 30 minutes in acetone to remove possible impurities from the surface of the polyurethane foam. A 0.1 M solution of FeCl_3_·6H_2_O was prepared in the ethanol. The dried foam was dipped into the 0.1 M FeCl_3_·6H_2_O solution and dried at 50 °C in the oven for 2 hours. The dried iron-coated foam was washed with water and ethanol to remove the excess loaded FeCl_3_·6H_2_O, which can later affect the hydrophobicity of the material. The washed Fe-coated foam was dried again at 50 °C in the oven for 2 hours. The catalyst-loaded polyurethane was exposed to 2.5 mL of pyrrole and kept for 2 hours. During that period, the pyrrole was catalyzed into polypyrrole. After polymerization, the yellow color of the polyurethane turned black. The polypyrrole-modified polyurethane was thoroughly washed with deionized water and ethanol successively. The washed and dried PP–PU was introduced into a 0.05 M solution of silicic acid in ethanol for 1 hour. The silicic acid-treated PP–PU (SiO_2_–PP–PU) was further treated in 0.05 M octadecyltrichlorosilane in toluene for 30 minutes. The ODTCS–SiO_2_–PP–PU was first dried at room temperature and then dried at 50 °C for 3 hours.

### 2.4 Oil adsorption, flux, desorption and emulsion separation experiment

The oil adsorption experiments were carried out by adding 50 mL of various non-polar organic solvents into a container. Prior to dipping into the oil, the weight of the ODTCS–SiO_2_–PP–PU was measured with the help of an electric balance. The oil adsorption experiment was performed by dipping the ODTCS–SiO_2_–PP–PU into the oil for 1 minute. During this time, the ODTCS–SiO_2_–PP–PU was pressed 3 times into the non-polar organic solvents to remove any trapped air from it. After that, the oil-saturated ODTCS–SiO_2_–PP–PU was immediately transferred to a container in the weighing balance to measure its mass. The weight gain ratios after adsorption of the targeted solvents were calculated using the following equation:

1 *W*% = (*M*_1_ − *M*_0_)/*M*_0_ × 100

where *W*% is the weight gain ratio of ODTCS, *M*_1_ is the weight of the ODTCS–SiO_2_–PP–PU after absorption, *M*_0_ is the weight of the ODTCS–SiO_2_–PP–PU prior to adsorption of the oil or organic solvents.

The flux was found by passing the hexane through the tube containing ODTCS–SiO_2_–PP–PU under gravity. The flux was calculated using the following equation:

2 Flux = *V*/*A* × *t*

where *V* is the volume (L), *A* is the area (m^2^) and *t* is the time (h).

The surfactant-free and surfactant (dodecylbenzene sulfonic acid) stabilized emulsion of water in oil was prepared by adding 1 mL of water into 8 mL of chloroform, then 1 mg of the surfactant was added. In the case of surfactant-free emulsion, the water and chloroform were sonicated for 30 minutes to stabilize the emulsion before passing through the ODTCS–SiO_2_–PP–PU. The surfactant stabilized emulsions were developed using dodecylbenzene sulfonic acid.

The desorption experiment was performed by squeezing the foam that released the adsorbed non-polar liquids. In the case of highly volatile liquids such as hexane or petrol, the ODTCS–SiO_2_–PP–PU was kept in the fume hood until dry. In the case of viscous non-polar organic solvents, after releasing as much of the liquid as possible, the viscous liquid stuck to the walls of the ODTCS–SiO_2_–PP–PU. The sticky viscous liquid was removed by dipping in hexane with multiple squeezing cycles and then washing with ethanol. After that, the washed ODTCS–SiO_2_–PP–PU was kept in the fume hood to dry.

## 3. Results and discussion

### 3.1 Polyurethane surface functionalization

**Scheme 1:**
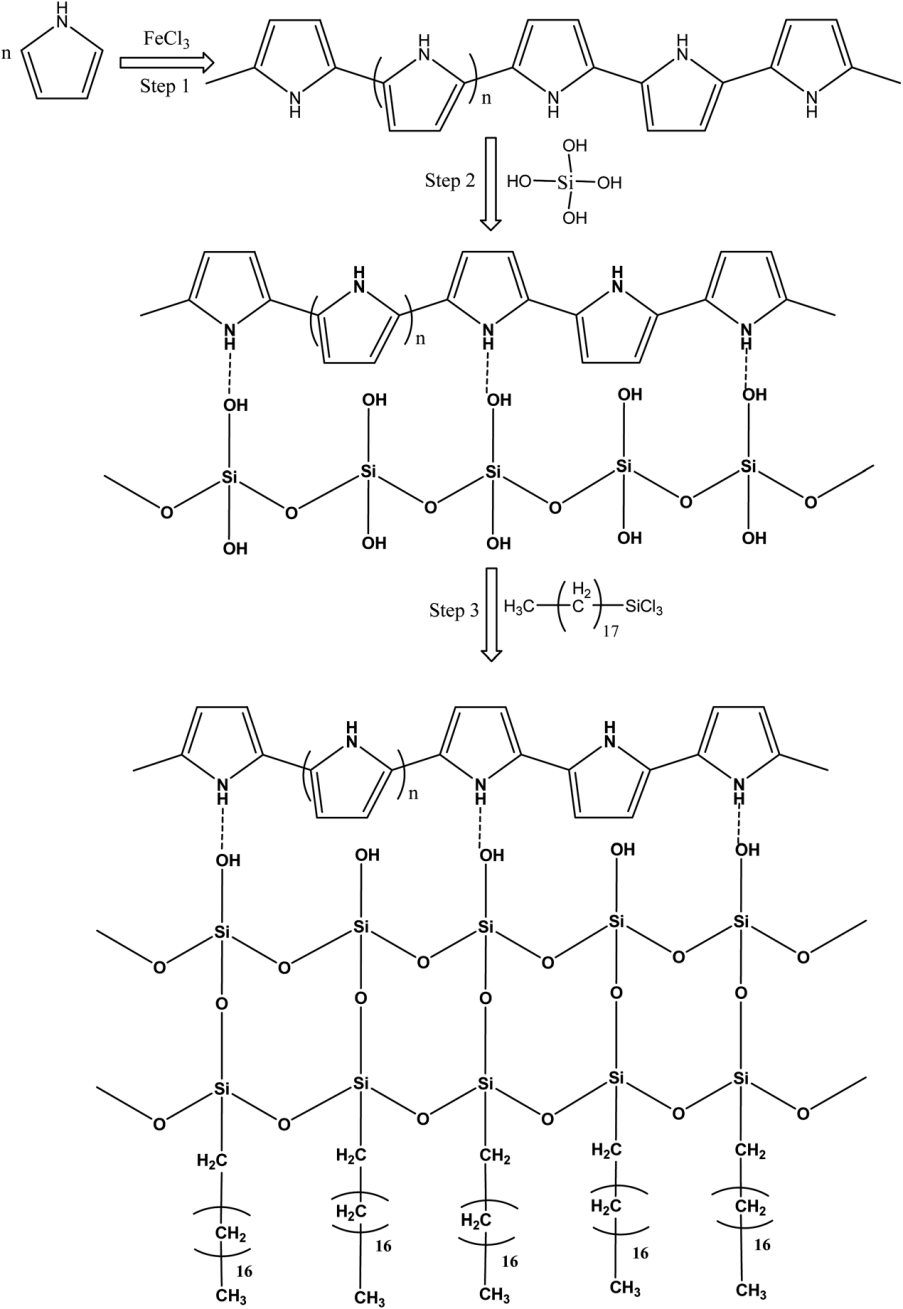
Schematic illustration of the reactions taking place between pyrrole, silicic acid and ODTCS on the surface of the polyurethane.

Pure polyurethane lacks specific wettability. By modifying the surface of PU at different steps, different surface wettability was displayed according to the presence of the surface functionalities. During absorption of the liquid, it showed the capacity to absorb both oil and water. Due to this observation, PU cannot be used directly for oil and water separation applications. The surface functionalization is a crucial step to obtain a stable material with a specific surface wettability. In the first step, a polypyrrole network was generated on the polyurethane by pyrrole polymerization. For polymerization, Fe^3+^ acts as a catalyst to catalyze pyrrole (C_4_H_4_NH) polymerization into polypyrrole.^20^ The ferric catalyst was introduced into the walls of the polyurethane where it facilitated rapid polymerization of the pyrrole and assisted the strong adherence of the polypyrrole to the porous interconnected walls of the polyurethane. After polymerization, the color of the polyurethane turned black due to the presence of polypyrrole. Due to polymerization, the introduced amino group played a vital role in providing a stable surface for further functionalization. The silica was introduced by silicic acid. The silicic acid demonstrated strong intermolecular forces with the polypyrrole on the surface of polyurethane. The silicic acid built a second functionalized network on the polyurethane through polypyrrole. The silicic acid hydroxyl group reacted in the third stage with ODTCS, where the long chains of the octadecyl group could link with polyurethane through the silicon. The overall interaction and the reactions of pyrrole, silica, and ODTCS on the porous walls of polyurethane are illustrated in Scheme 1.

### 3.2 Characterization of the ODTCS–SiO_2_–PP–PU

**Fig. 1:**
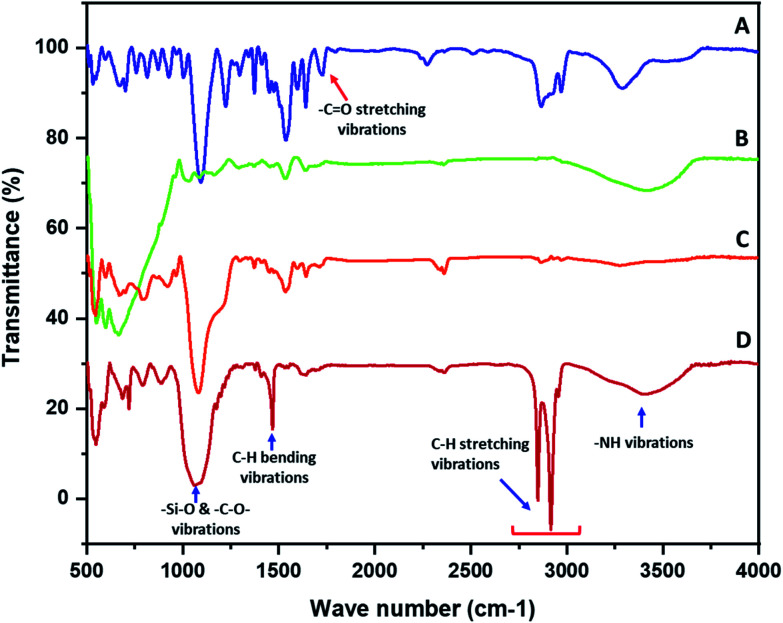
The FTIR spectra of (A) polyurethane, (B) PP–PU, (C) SiO_2_–PP–PU, and (D) ODTCS–SiO_2_–PP–PU.

The functionalization of the polyurethane at various stages was established with the help of FTIR. The FTIR spectra of the polyurethane prior to functionalization were collected and the characteristic absorption bands of polyurethane were observed. The polyurethane displayed a carbonyl (–C=O) stretching absorption band at 1726 cm^−1^. The –N–H deforming band appeared at 1537 cm^−1^ and the stretching band appeared at 3292 cm^−1^. The –C–H symmetric and asymmetric stretching vibrations were observed in the range of 2800 to 3000 cm^−1^.^21^ A sharp absorption band that appeared in the non-functionalized polyurethane at 1094 cm^−1^ was assigned to –C–O–C– stretching (Fig. 1A). The FTIR spectra of the polypyrrole-coated polyurethane was recorded and a broad absorption band was observed at 600 cm^−1^. The presence of a broad peak was due to the presence of ferric/ferrous components in the composite. A broad absorption peak after 3000 cm^−1^ was observed in the spectra of PP–PU. The appearance of a broad peak after 3000 cm^−1^ was attributed to the NH group of polypyrrole and polyurethane. Most of the absorption peaks in the PP–PU FTIR spectra appeared broad compared to those in the non-functionalized polyurethane spectra. Due to the broad absorption bands, most of the polypyrrole peaks were merged under the signal. The peaks appearing at 1292 cm^−1^ were assigned to the =C–H vibrations^22^ (Fig. 1B). The FTIR spectrum of SiO_2_–PP–PU revealed that the incorporation of silica into PP–PU was successful. A sharp absorption band appeared at 1081 cm^−1^ which represents the –Si–O–Si– antisymmetric stretching vibrations. The appearance of Si–O–Si bands in SiO_2_–PP–PU demonstrated the successful incorporation of SiO_2_ into PP–PU (Fig. 1C). In the case of, ODTCS–SiO_2_–PP–PU along with the –Si–O–Si– peaks, the more prominent sharp peaks of –C–H stretching vibrations were observed at 2849, 2916 and 2955 cm^−1^. The appearance of –Si–O–Si– and –C–H prominent sharp peaks confirmed the successful functionalization of SiO_2_–PP–PU with ODTCS (Fig. 1D).

**Fig. 2:**
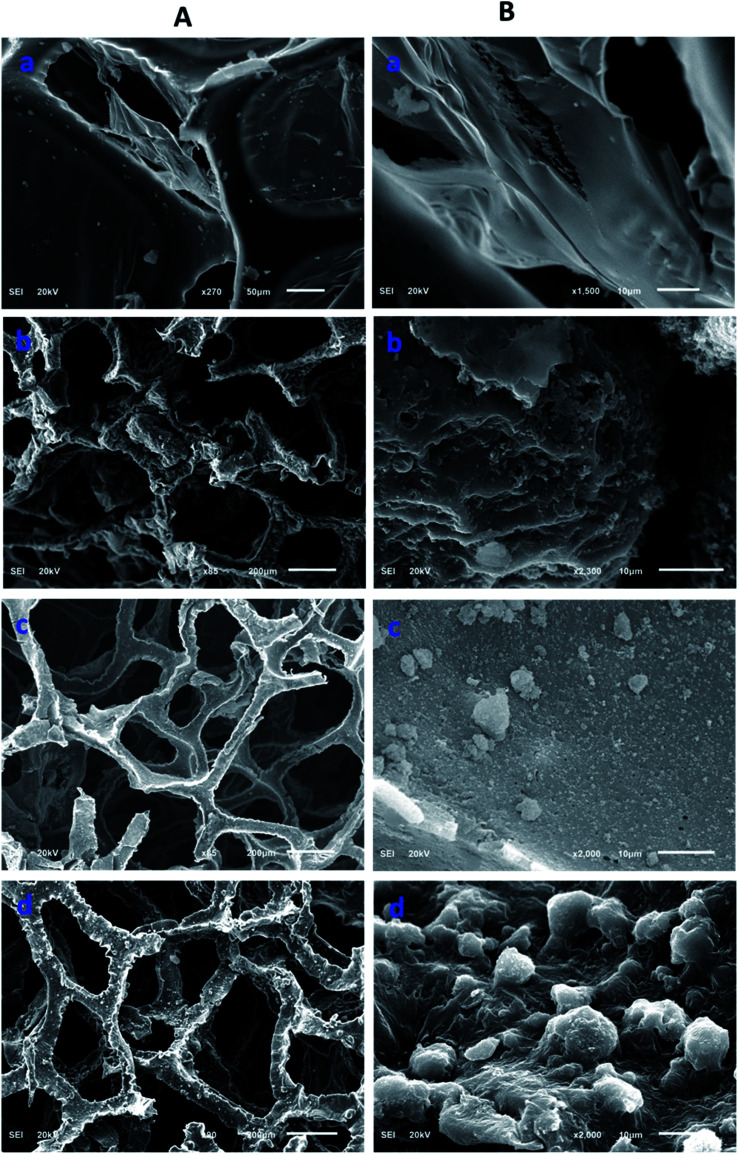
SEM images at two different magnifications (A and B) of (a) PU, (b) PP–PU, (c) SiO_2_–PP–PU and (d) ODCS–SiO_2_–PP–PU.

**Scheme 2:**
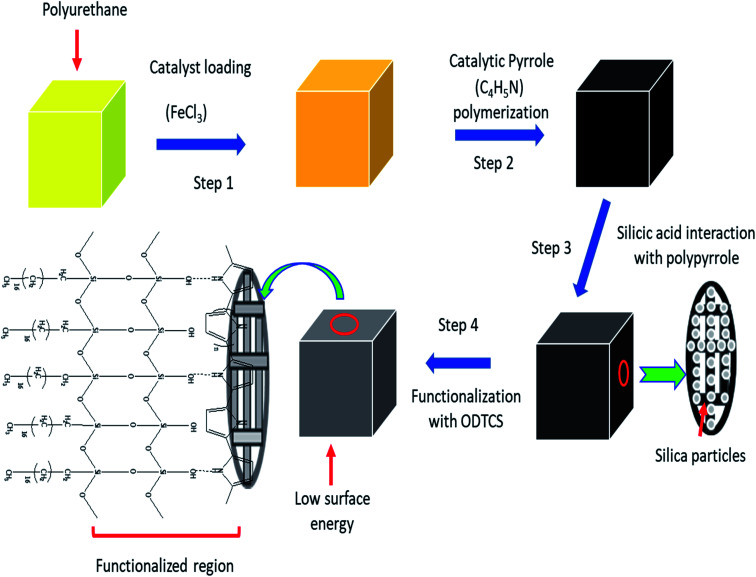
Schematic showing the generation of functionalized polyurethane by the combination of polypyrrole, silica, and ODTCS on the polyurethane walls.

Morphological information of various forms of the modified polyurethane was collected by scanning electron microscopy (SEM). The SEM images provided useful information related to the surface changes after each step of modification. The polyurethane consists of a porous network. The walls of the PU appeared planer and this was evident from the SEM images of the unmodified PU (Fig. 2A(a) and B(b)). The polymerization of pyrrole on the planar walls of the polyurethane was clearly observed by SEM. The walls of the polyurethane appeared wavier and thicker after the catalytic polymerization of the pyrrole. The polyurethane maintained a porous network after the pyrrole polymerization on the walls of the polyurethane, as shown in Fig. 2A(b). SEM images have revealed the SiO_2_ distribution on the walls of the polypyrrole-modified polyurethane. Apart from the uniform distribution of the SiO_2_, some aggregates of SiO_2_ were also observed (Fig. 2A(c) and B(c)). In the case of ODTCS–SiO_2_–PP–PU, the SEM images display the sheets which cover the SiO_2_-incorporated pyrrole-polymerized walls of the polyurethane. The sheets appeared as a result of ODTCS and even a lump of the SiO_2_ was also covered under the sheet (Fig. 2A(d) and B(d)). The SEM images revealed that the surface morphology of the polyurethane walls changed after each step of modification (Scheme 2).

**Fig. 3:**
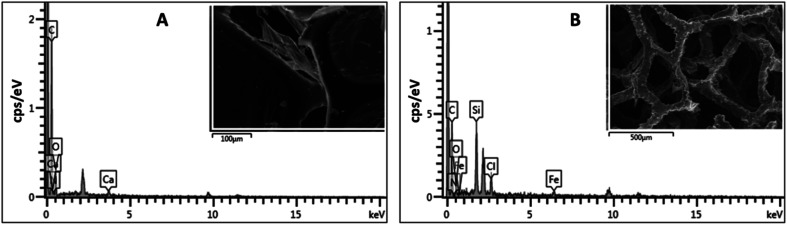
EDX spectra of (A) PU and (B) ODTCS–SiO_2_–PP–PU.

The 3D porous network was further investigated using EDX spectroscopy. EDX spectroscopy is a valuable analytical tool that provides elemental information on the material. The EDX spectrum of ODTCS–SiO_2_–PP–PU displayed additional peaks of Si, Cl, and Fe that were absent in the EDX spectrum of PU (Fig. 3). The sharp peak of Si highlights the presence of SiO_2_ and the silane group. Ferric chloride was loaded into the polyurethane to catalyze the polymerization reaction of pyrrole. Due to this, Fe and Cl peaks appear in the ODTCS–SiO_2_–PP–PU EDX spectrum. The appearance of the relevant elements during the EDX analysis support the successful production of the 3D porous composite of polyurethane.

### 3.3 Specific surface wettability of ODTCS–SiO_2_–PP–PU

**Fig. 4:**
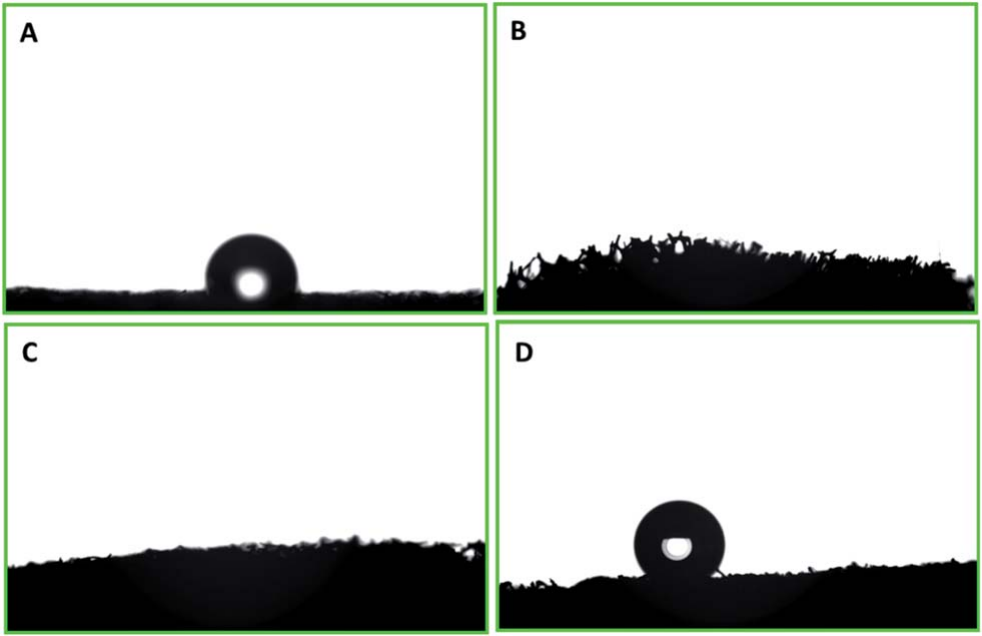
Water contact angle on the surfaces of (A) PU, (B) PP–PU, (C) SiO_2_–PP–PU and (D) ODTCS–SiO_2_–PP–PU.

The surface wettability of PU was evaluated after each step of modification and functionalization. The polyurethane displayed a water contact angle of 109.6° ± 2.3°. Water droplets have also shown a tendency to adhere to the surface. PP–PU wettability was entirely changed, and the water droplet was readily adsorbed by the surface. This change of the surface behavior was due to the presence of numerous NH– groups of polypyrrole that impart the polar behavior to the surface. The same behavior was observed for the SiO_2_–PP–PU. In the case of SiO_2_–PP–PU, the polarity still dominated the surface of polyurethane. The surface of ODTCS–SiO_2_–PP–PU became superhydrophobic after the introduction of the low surface energy of ODTCS functionalization that was stabilized on the walls of the polyurethane with the help of the silica and polypyrrole. The superhydrophobicity of ODTCS–SiO_2_–PP–PU was revealed by the water contact angle of 154.7° ± 0.8°. The adherence of the water droplet that was observed on the unmodified polyurethane disappeared in the case of ODTCS–SiO_2_–PP–PU. The water droplet from the auto-controller micropipette continuously touched the surface of ODTCS–SiO_2_–PP–PU by bringing the auto-controller micropipette down, but the surface did not shown affinity towards the water droplet. The high-water contact angle revealed that after functionalization of the SiO_2_–PP–PU with ODTCS, the surface energy was substantially decreased, which was evident from the three-dimensional macroporous surface behavior towards the water (Fig. 4).

### 3.4 Evaluation of the absorption, regeneration and emulsion separation capability

The ODTCS–SiO_2_–PP–PU absorption capacity and its regeneration capability were evaluated for oils and various non-polar organic pollutants. The macroporous superhydrophobic network of ODTCS–SiO_2_–PP–PU displayed a good absorption capability for a range of non-polar organic liquids. The absorption capacity was evaluated for the range of non-polar organic solvents and petrol components including hexane, heptane, iso-octane, dodecane, petrol, cyclohexane, *o*-xylene, and toluene.

**Fig. 5:**
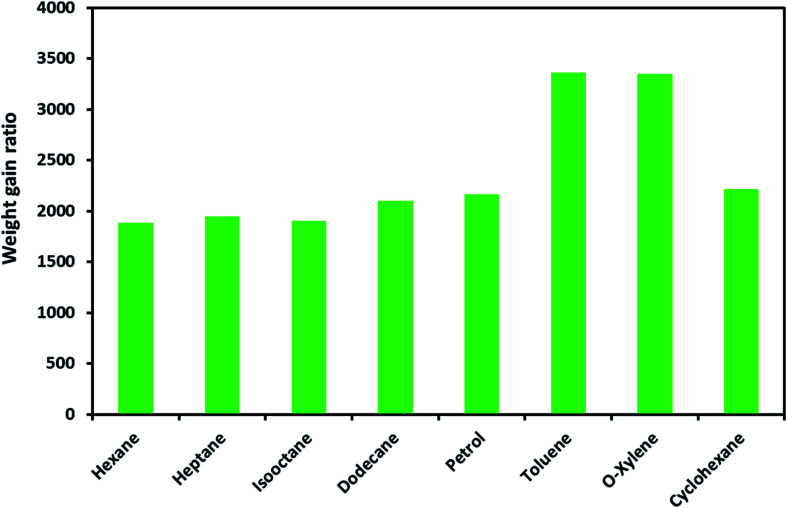
Absorption capacity of ODTCS–SiO_2_–PP–PU for oil and non-polar organic solvents.

The weight gain ratio by using eqn (1) was found in the range of 1800 to 3400% (Fig. 5). The absorption capacity for petrol was found at 2162%. It is crucial to note that the absorption capacity might predominantly depend on the density of the various oils and the organic solvents.^23^ The developed 3D macroporous superhydrophobic network of ODTCS–SiO_2_–PP–PU displayed an absorption capacity in the range of 18 to 34 times better than that of the previously reported recent materials, including aerogel composites (2–16 times),^24^ PDMS-SW (12–27 times),^25^ magnetic silicone sponges (7–17 times),^26^ PDMS sponges (4–11 times),^23^ and nitrogen-rich carbon aerogels (6–11 times).^27^ Moreover, toluene absorption was specifically compared with the previous literature and demonstrated better efficiency here than in the previously reported work (Table 1). The increase in weight gain ratio is evidence that ODTCS–SiO_2_–PP–PU has an excellent capability to absorb a large quantity of non-polar organic liquids and spilled oils. Moreover, its facile and robust route of fabrication using cost-effective raw materials is an attractive feature for scale-up and readily deployable for application.

**Table 1:** Comparison of the absorption capacity for toluene by the ODTCS–SiO_2_–PP–PU to the reported materials

Sr.#	Superhydrophobic material	Preparation methods	Absorption capacity (toluene)	Regeneration	Ref.
1	Monolithic superhydrophobic silica aerogel	Sol–gel process	9	Distillation and vacuum filtration	28
2	Porous BNNS/PVDF composite material	Gelation and freeze-drying process	5	Washing in ethanol and drying at 60 °C in air	29
3	Magnetic graphene foam	Hummer's method	19	Hexane immersion	30
		Gas based reduction			
		Co-precipitation			
4	Fluorinated polydopamine/chitosan/reduced graphene oxide composite aerogel	Hummers' method	8	Heating and squeezing	31
		Hydrothermal			
		Immersion			
		Fluorination			
5	Graphene foam	Modified hummers method	20	Washing in ethanol and oven drying	32
		Sol–gel method			
		Hydrolyzed and curing			
6	PDMS sponge	Sugar templating process	5	Squeezing	23
7	Porous BN nanosheets	Dynamic templating approach	∼24	Burning and heating in air	33
8	Superhydrophobic/superoleophilic cotton fiber	Sol–gel process	∼30	Drained under mild suction by a vacuum air pump	34
		Self-assembling			
9	Carbon aerogel	Hydrothermal and post-pyrolysis process	29	By heating	35
10	PDMS-SW	Immersion	11.5	Mechanical squeezing	25
11	Magnetic silicone sponge	Hydrolysis and polymerization	9	By squeezing	26
12	ODTCS–SiO_2_–PP–PU	Catalytic polymerization	34	By squeezing	This work
		Immersion			

**Fig. 6:**
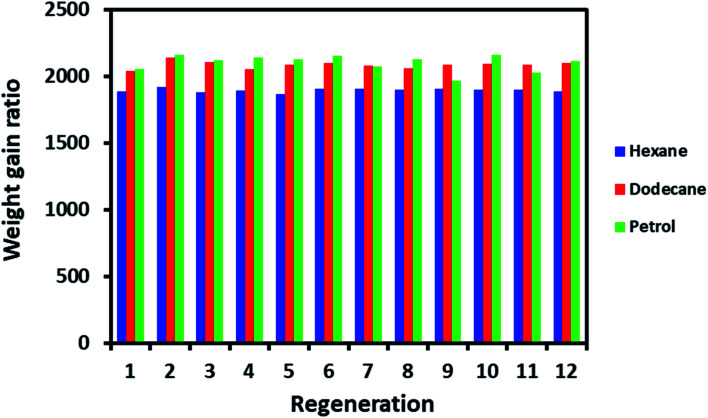
Regeneration evaluation of ODTCS–SiO_2_–PP–PU for hexane, dodecane, and petrol.

The regeneration is a crucial factor in deciding the fate of material scalability and practical application. For example, cost-effective materials have lost attention for practical use if they do not display the capability to regenerate after a single use. ODTCS–SiO_2_–PP–PU regeneration was evaluated with hexane, dodecane, and petrol. The same ODTCS–SiO_2_–PP–PU was used for the regeneration study of various organic pollutants. ODTCS–SiO_2_–PP–PU displayed excellent regeneration capabilities for the analyzed hexane, dodecane and petrol with a RSD of ±0.7 (*n* = 12), ±1.2 (*n* = 12) and ±2.9 (*n* = 12), respectively (Fig. 6). The spongy nature of the macroporous material facilitated oil removal by squeezing it out. After multiple uses, the surface maintained its superhydrophobicity and repelled the water strongly from its surface. The regenerated ODTCS–SiO_2_–PP–PU was further investigated by collecting its FTIR spectrum. The FTIR spectrum of the regenerated ODTCS–SiO_2_–PP–PU revealed all the characteristics peaks that were present in the modified PU before its use (Fig. S1†). This regeneration behavior has revealed the robustness of the material and that it can be used for a long time.

The capability of ODTCS–SiO_2_–PP–PU was evaluated for the separation of the surfactant-free and surfactant-stabilized emulsion. The surfactant-free and the surfactant-stabilized water in oil emulsion were prepared by using distilled water and chloroform. The surfactant-free emulsion was stabilized by sonication whereas the surfactant-stabilized emulsion was prepared by adding the surfactant. The development of the emulsions could be observed in their respective vials that appeared as a milky color (Fig. S2 and S3†). Emulsions were passed through the ODTCS–SiO_2_–PP–PU that were tightly packed into the nozzle of the apparatus. The ODTCS–SiO_2_–PP–PU permitted the chloroform to pass while preventing the water from passing. It is shown in Fig. S2 and S3.†

**Fig. 7:**
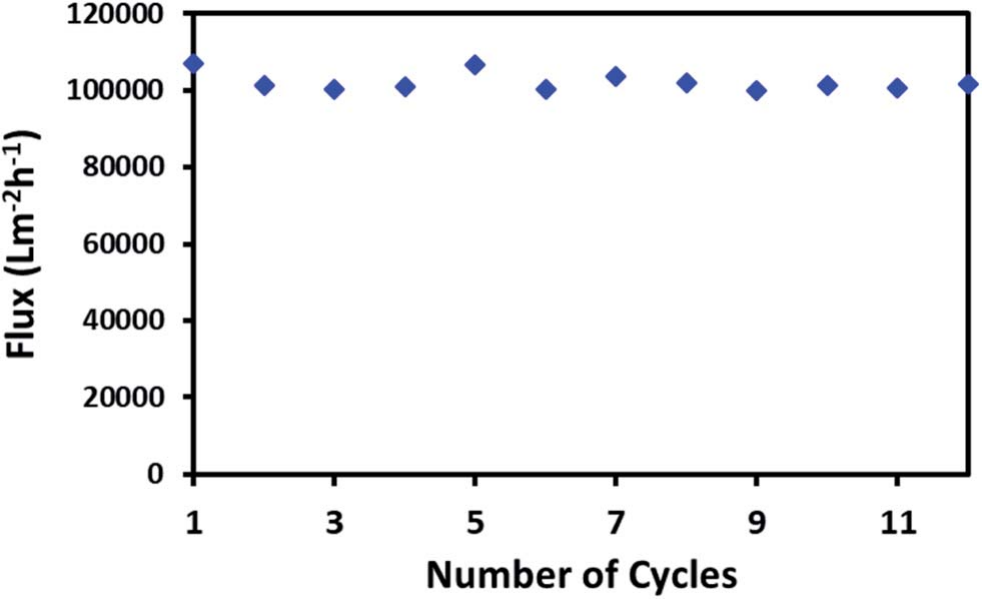
Gravity-driven passage of hexane flux from ODTCS–SiO_2_–PP–PU under ambient conditions.

High flux through a continuous network of absorbent material is an important factor for the porous material in order to absorb large spills of oil or non-organic solvents. The macroporous network of ODTCS–SiO_2_–PP–PU displayed an outstanding capability for the continuous passing of the oil or non-polar organic component. Through the porous network, the non-polar component passed very fast while the water was rejected. The flux for hexane was found to be 102 068 Lm^−2^ h^−1^. This flux value for the non-polar organic component is exceptionally high. As depicted in Fig. 7, the hexane flux was evaluated 12 times and the RSD was found to be ±2.31. This indicates that after multiple uses, the porous network of ODTCS–SiO_2_–PP–PU was not clogged and continuously allowed a high flux of hexane through it. The flux is high compared to values in previously reported flux studies (Table 2). M. H. Tai studied a SiO_2_-carbon composite membrane with a hexane flux of 2648.8 Lm^−2^ h^−1^ under gravity and a contact angle of 144.2°.^36^ In the synthesized ODTCS–SiO_2_–PP–PU, the hexane flux (102 068 Lm^−2^ h^−1^) was greater and displayed more surface hydrophobicity with a contact angle of 154.7° ± 0.8°. The macroporous network provided fewer coagulation chances, which are common issues with the membranes used for oil/water separation.

**Table 2:** The comparison of the hydrophobicity and the flux of ODTCS–SiO_2_–PP–PU with other reported materials

Sr.#	Superhydrophobic material	Preparation methods	Contact angle	Flux (Lm^−2^ h^−1^)	Ref.
1	PVDF–HFP membrane	Electrospinning	134.0 ± 3.2°	94 000 (gasoline)	37
2	SiO_2_–carbon composite membrane	Electrospinning	144.2 ± 1.2°	2648.8 (hexane)	36
3	TPU microfiber membrane	Force spinner	NA	4659 (oil flux)	38
4	TPU-PNIPAM membrane	Force spinner	NA	503 (oil flux)	38
		Free radical polymerization			
5	PAA-*g*-PVDF membrane	Salt-induced	NA	2320 (hexadecane/H_2_O)	39
		Phase inversion			
		60 Co γ-ray source irradiation			
6	ODTCS–SiO_2_–PP–PU	Catalyst based polymerization	154.7° ± 0.8°	102 068 (hexane)	This work

**Fig. 8:**
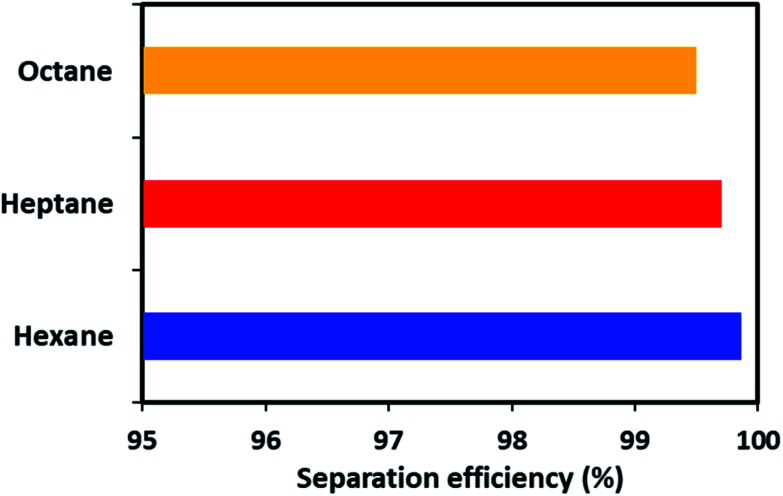
Separation efficiency of the ODTCS–SiO_2_–PP–PU for various organic solvents from water.

Apart from the gravity-based separation, the dynamic separation of oil/water was also evaluated by applying pressure with the help of a peristaltic pump. For this purpose, ODTCS–SiO_2_–PP–PU was placed at the interface of the oil and water in such a way that it was more than half dipped into the water (Video S1†). This strategy was adopted to observe during oil passage whether the water passed or was prevented from passing by the superhydrophobic nature of the material. In the dynamic separation analysis, it was observed that hexane passed rapidly from the ODTCS–SiO_2_–PP–PU network, while the water was completely prevented from passing. This is evidence that the surface remained superhydrophobic during the separation of the non-polar organic and water mixture. Through the ODTCS–SiO_2_–PP–PU, various systems of hexane/water, heptane/water and octane/water were separated to evaluate the separation efficiency of the porous network of ODTCS–SiO_2_–PP–PU. The separation efficiency for the hexane, heptane and octane were found in the range of 99.5 to 99.8% (Fig. 8). The efficient separation can be explained by the superhydrophobic porous network that spread over all of the polyurethane walls. It rapidly allowed the passage of the non-polar component and completely prevented the passage of the water through it.

## 4. Conclusions

In this work, a cost-effective combination of ODTCS, SiO_2_, polypyrrole, and polyurethane was used to develop a superhydrophobic porous network for the efficient separation of spilled non-polar organic contaminants from water. The surface functionalization of the polyurethane substantially improved the contact angle from 109.6° ± 2.3° to 154.7° ± 0.8°. Its superhydrophobic porous network rapidly passed non-polar organic liquids and displayed an extraordinarily high flux of 102 068 Lm^−2^ h^−1^. The porous three-dimensional hydrophobic network has a great capacity to absorb and keep the non-polar organic solvents. It displayed an absorption capacity of 34 times its weight for toluene under ambient conditions. It can be used multiple times and the absorbed oil is recovered from ODTCS–SiO_2_–PP–PU by squeezing it. ODTCS–SiO_2_–PP–PU displayed good mechanical stability and after squeezing, the porous interconnected network was not destroyed. It displayed excellent separation efficiency for various organic solvents in the range of 99.5 to 99.8%, good recyclability, a facile route of synthesis, high flux, and a good absorption capacity. It displayed an excellent regeneration capability with hexane, dodecane, and gasoline. The RSD was found in the range of ±0.7 to ±2.9 after 12 cycles of use. Due to the high flux, great stability, excellent absorption capacity, and an efficient separation capability of oil from water and water in oil emulsions, ODTCS–SiO_2_–PP–PU is a unique material for the separation of oil from water.

## Conflicts of interest

There are no conflicts to declare.

## Supplementary Material

RA-010-C9RA06579B-s001

RA-010-C9RA06579B-s002
