# Supplementary material for: A flexible biomimetic superhydrophobic and superoleophilic 3D macroporous polymer-based robust network for the efficient separation of oil-contaminated water
Source: RSC Adv. 2020 Jan 31;10(9):5088–97. doi: 10.1039/c9ra06579b (PMC9049064; doi:10.1039/c9ra06579b)
Supplement: RA-010-C9RA06579B-s001 [file RA-010-C9RA06579B-s001.pdf]

Electronic Supplementary Material (ESI) for RSC Advances.
This journal is © The Royal Society of Chemistry 2019

Supplementary file

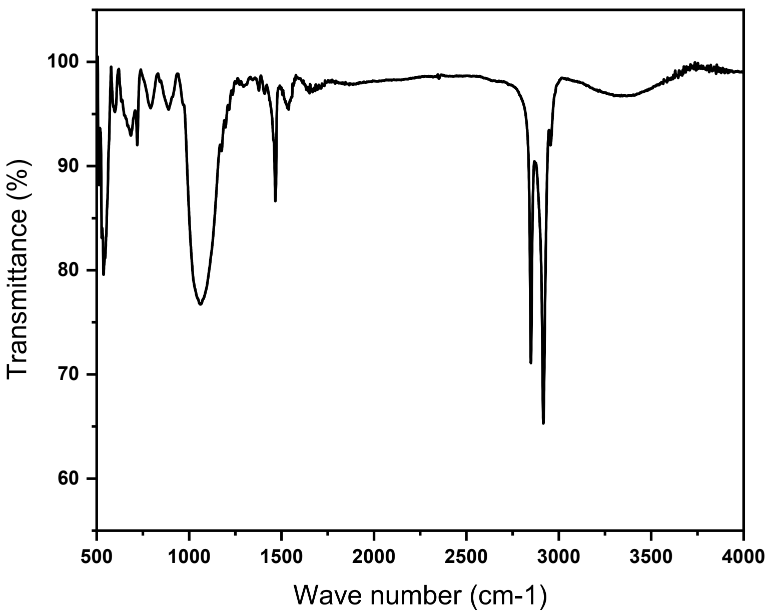

Fig. S1 FTIR spectra of the regenerated ODTCS-SiO_2-PP-PU

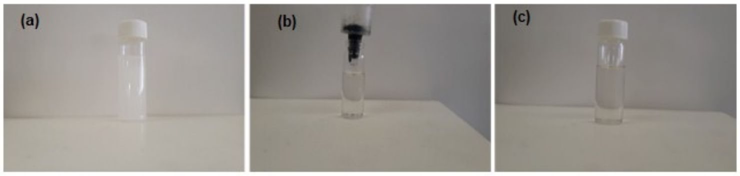

Fig. S2 Separation of surfactant free water in oil emulsion (A) water in oil emulsion, (B) Separation process, (C) Separated oil from water by using the ODTCS-SiO_2-PP-PU

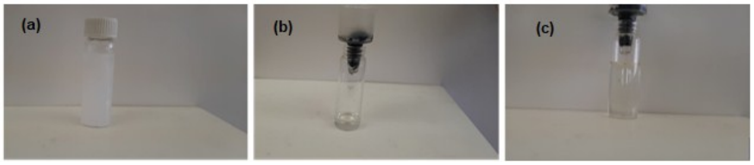

Fig. S3 Separation of surfactant stabilized water in oil emulsion (A) water in oil emulsion, (B) Separation process, (C) Separated oil from water by using the ODTCS-SiO_2-PP-PU
